# Key roles of autophagosome/endosome maturation mediated by Syntaxin17 in methamphetamine-induced neuronal damage in mice

**DOI:** 10.1186/s10020-023-00765-9

**Published:** 2024-01-03

**Authors:** Xi Wang, Miaoyang Hu, Jingrong Chen, Xinyu Lou, Hongchao Zhang, Muhan Li, Jie Cheng, Tengfei Ma, Jianping Xiong, Rong Gao, Xufeng Chen, Jun Wang

**Affiliations:** 1 Key Lab of Modern Toxicology (NJMU), Department of Toxicology, School of Public Health, Ministry of Education, Nanjing Medical University, 101 Longmian Street, Nanjing, Jiangsu 211166 China; 2 School of Pharmacy, Nanjing Medical University, 101 Longmian Street, Nanjing, Jiangsu 211166 China; 3 Department of Hygienic Analysis and Detection, Key Laboratory of Modern Toxicology, School of Public Health, Ministry of Education, Nanjing Medical University, Nanjing, China; 4 Department of Emergency Medicine, the First Affiliated Hospital of Nanjing Medical University, 300 Guangzhou Road, Nanjing, Jiangsu 210029 China; 5 China International Cooperation Center for Environment and Human Health, Nanjing Medical University, 101 Longmian Street, Nanjing, Jiangsu 211166 China

**Keywords:** Methamphetamine, Syntaxin 17, Autophagy, Autophagosome, Endosome

## Abstract

**Background:**

Autophagic defects are involved in Methamphetamine (Meth)-induced neurotoxicity. Syntaxin 17 (Stx17), a member of the SNARE protein family, participating in several stages of autophagy, including autophagosome-late endosome/lysosome fusion. However, the role of Stx17 and potential mechanisms in autophagic defects induced by Meth remain poorly understood.

**Methods:**

To address the mechanism of Meth-induced cognitive impairment, the adenovirus (AV) and adeno-associated virus (AAV) were injected into the hippocampus for stereotaxis to overexpress Stx17 in vivo to examine the cognitive ability via morris water maze and novel object recognition. In molecular level, the synaptic injury and autophagic defects were evaluated. To address the Meth induced neuronal damage, the epidermal growth factor receptor (EGFR) degradation assay was performed to evaluate the degradability of the “cargos” mediated by Meth, and mechanistically, the maturation of the vesicles, including autophagosomes and endosomes, were validated by the Co-IP and the GTP-agarose affinity isolation assays.

**Results:**

Overexpression of Stx17 in the hippocampus markedly rescued the Meth-induced cognitive impairment and synaptic loss. For endosomes, Meth exposure upregulated Rab5 expression and its guanine-nucleotide exchange factor (GEF) (immature endosome), with a commensurate decreased active form of Rab7 (Rab7-GTP) and impeded the binding of Rab7 to CCZ1 (mature endosome); for autophagosomes, Meth treatment elicited a dramatic reduction in the overlap between Stx17 and autophagosomes but increased the colocalization of ATG5 and autophagosomes (immature autophagosomes). After Stx17 overexpression, the Rab7-GTP levels in purified late endosomes were substantially increased in parallel with the elevated mature autophagosomes, facilitating cargo (Aβ42, p-tau, and EGFR) degradation in the vesicles, which finally ameliorated Meth-induced synaptic loss and memory deficits in mice.

**Conclusion:**

Stx17 decrease mediated by Meth contributes to vesicle fusion defects which may ascribe to the immature autophagosomes and endosomes, leading to autophagic dysfunction and finalizes neuronal damage and cognitive impairments. Therefore, targeting Stx17 may be a novel therapeutic strategy for Meth-induced neuronal injury.

**Supplementary Information:**

The online version contains supplementary material available at 10.1186/s10020-023-00765-9.

## Introduction

Amphetamine-type stimulants (ATSs) have become one of the most widely abused drugs worldwide. According to the 2021 World Drug Report, the number of people who used ATSs in 2019 reached 27 million (UNDODC 2021). Methamphetamine (Meth), commonly known as “ice”, is a typical representative of ATS. Due to its strong neurotoxicity, Meth exposure leads to a series of deteriorating effects on the brain. Research has shown that it promotes the aggregation of β-amyloid and p-tau proteins and causes synaptic loss, all of which are the main characteristics of neurodegenerative diseases (Shukla et al. 2019; Li et al. 2018). Therefore, amelioration of pathological protein deposition and synaptic loss is critical and may provide an effective therapeutic avenue for Meth-induced neuronal damage.

Autophagy is a pivotal cellular metabolic pathway that degrades damaged or aged molecules such as organelles and proteins and provides the necessary nutrients and energy for cell survival. This process plays a particularly critical role in clearing pathological proteins and harmful metabolic byproducts in neurons, maintaining neural homeostasis (Menzies et al. 2015; Nixon 2013). Meth exposure has been shown to disturb the autophagic process. After Meth treatment, the astrocytic cell line SVGA exhibited potent elevation of ATG5, ATG7, Beclin-1 and LC3, and inhibition of autophagy exacerbated astrocytic damage (Cao et al. 2016). In addition, DNA damage-inducible transcript 4 (DDIT4), a factor involved in Meth-induced damage in dopaminergic neuronal cells, was confirmed to have a positive regulatory effect on autophagy-related proteins (Li et al. 2017). Additionally, excessive expression of LC3 and ATG5 was observed in postmortem Meth abusers, whose brains are characterized by neurodegenerative changes (Khoshsirat et al. 2020). This evidence directly demonstrates the neural toxicity involving autophagic defects induced by Meth; however, the underlying mechanism is not yet fully understood.

The maturation of endosomes and autophagosomes is a crucial upstream event in the autophagic process, and its completion is important for autophagic function. Endosomes include different subgroups, such as early endosomes, late endosomes, and multivesicular bodies. Early endosomes are mainly composed of vesicle membranes and lumens, which participate in cell uptake and endocytosis, while late endosomes are formed by the fusion of early endosomes and contain a variety of hydrolytic enzymes that can hydrolyze and decompose cargos in endocytic vesicles and the lumen of endosomes (Hyttinen et al. 2013; Kimura et al. 2007). The process of endosome maturation not only involves the fusion and differentiation of endosomal membranes but also impacts a series of signal transduction pathways. For example, Rab GTPase family proteins act as molecular switches to participate in membrane fusion and differentiation during endosome maturation, while glycoproteins and acid hydrolases regulate the specificity and function of endomembrane surfaces. Abnormal or defective endosomal maturation is closely associated with the development of neurodegenerative diseases, such as Alzheimer’s disease (AD), Parkinson’s disease (PD), Lewy body dementia, amyotrophic lateral sclerosis (ALS), and hereditary spastic paraplegia (HSP) (Kaur et al. 2018, Luzio et al. 2010, Huotari et al. 2011, Huotari et al. 2012, Schreij et al. 2016, Sun et al. 2010). The fusion between autophagosomes and late endosomes/lysosomes is a crucial regulatory step ensuring the degradation of autophagic cargos. Of note, Syntaxin 17 (Stx17), a membrane protein belonging to the SNARE protein family, is pivotal to mediating membrane fusion of vesicles, allowing the transport and exchange of substances between different organelles. Notably, our previous studies showed that Meth exposure leads to a dramatic decrease in Stx17 in neuronal cells, which in turn impedes autophagosome-late endosome/lysosome fusion, finalizing pathological protein accumulation in vesicles (Kumar et al. 2018; Xu et al. 2021; Zhu et al. 2022); however, whether Stx17 participates in autophagosome and endosome maturation in Meth induced neurotoxicity to date remains poorly understood.

Therefore, in the current study, the potential effects of Stx17 on the modulation of autophagosome/endosome maturation were examined, with the aim of addressing the underlying mechanisms of Meth-induced vesicle fusion defects and concomitant neurodegenerative-like changes and behaviors, which may provide insights into potential strategies for Meth-induced neurotoxicity.

## Materials and methods

### Reagents

Meth was obtained from the National Institutes for Food and Drug Control (Beijing, China), and the following antibodies were used: anti-LC3A/B (12,741 S), anti-Rab7 (9376T), and anti-Rab5 (3547T) antibodies were from Cell Signaling Technology; anti-p62 (ab209012), anti-Stx17 (ab229646), anti-ATG5 (ab108327), anti-EGFR (ab52894) and anti-Tau 214 (ab170892) were purchased from Abcam; anti-CCZ1 (sc-51,429) and anti-Flag (sc-7392) antibodies were obtained from Santa Cruz Technology; anti-PSD95 (20665-1-1AP) and anti-Stx17 (17815-1-AP) were from Proteintech. The HRP-conjugated secondary antibodies were from Biosharp. Alexa Fluor™ 594 goat anti-mouse IgG (A-11,005), Alexa Fluor™ 488 goat anti-rabbit IgG (A-11,008), Alexa Fluor™ 568 goat anti-mouse (A-11,011), and Alexa Fluor™ 405 goat anti-rabbit IgG (A-31,556) were purchased from Thermo Fisher Scientific.

### Mouse feeding

Wild-type C57BL/6J male mice were obtained from the Experimental Animal Center of Nanjing Medical University. The mice were housed in cages under controlled environmental conditions (temperature: 18 ~ 22 °C, humidity: 30 ~ 50%) with a 12:12 h dark/light cycle. All experiments were conducted under the control of the Ethics Committee of Animal Care and Experimentation of Europe and approved by the Institutional Animals Care and Use Committee (IACUC) at Nanjing Medical University (Approval No. 2010012-1).

### Stereotaxic microinjection of adeno-associated virus

Adeno-associated virus (AAV) was injected bilaterally into the hippocampus of 8-week-old mice. Brain stereotactic locators were purchased from RWD Life Science. rAAV-hsyn-Stx17-2 A-EGFP-WPRE-hGH (AAV-Stx17) and the empty vector rAAV-hsyn-EGFP-WPRE-hGH (AAV-VE) were built by BrainVTA. Mice were anesthetized with isoflurane and then fixed on a stereotaxic frame, and an incision was made along the midline of the head to expose the underlying skull. The bregma and posterior coordinates were used as the reference points. A total of 0.6 µl of the virus was injected bilaterally into the hippocampus of mice. The coordinates for the CA1 of the hippocampus were anterior/posterior (A/P): +2.1 mm and medial/lateral (M/L): 1.7 ± mm from and dorsal/ventral (D/V): 2.1 mm from the skull surface. The coordinates for the CA3 of the hippocampus were anterior/posterior (A/P): + 2.9 mm and medial/lateral (M/L): ± 3 mm from and dorsal/ventral (D/V): 3.8 mm. After injection, the injector was left in place for 6 min for the diffusion of AAV. Twenty-eight days after surgery, AAV-microinjected mice were employed for behavior tests. We categorize the experimental group receiving AAV-VE injections as the “Con + VE” group; the group receiving concurrent injections of Meth and AAV-VE is designated as the “Meth + VE” group; and the group receiving combined injections of Meth and AAV-Stx17 is defined as the “MS” group. These group names will be reflected in the corresponding figures.

### Behavioral tests

For each test, mice were habituated in the testing room for 2 h. Each apparatus was cleaned with 75% ethyl alcohol before testing to prevent potential bias due to olfactory cues.

### Morris water maze

The Morris water maze was used to evaluate spatial learning memory. The pool was filled with water (60 cm deep, 22–24 °C) and made opaque. The escape platform was placed in the northeast quadrant and submerged 2 cm under the water. Mice were trained for 4 days with 4 trials/day (60 s/trial). A training trial was completed when the mouse mounted and remained on the platform for 2 s or spent 60 s in the pool. After each trial, the mouse remained on the platform for an additional 30 s. If the animals could not find a platform within 60 s, they were placed on the platform for 30 s. During the probe trial on Day 5, the platform in the target quadrant was removed from the pool, and each mouse was given 60 s to navigate the pool.

### Novel object recognition test

The novel object recognition test was used to evaluate nonspatial recognition memory. In the familiarization phase, each mouse was allowed to explore the open-field recognition box with identical objects located in opposite and equidistant positions for 5 min. After a 1 h retention interval, the mouse returned to the open-field recognition box with two objects, the initial object explored during the familiarization phase and a newly introduced novel object with a different shape and color. For the discrimination phase, a mouse was allowed to explore for 5 min, and the time spent exploring each object was recorded. The discrimination index was calculated according to the following formula: discrimination index (time spent on novel objects/total time spent on both objects) ×100%. Exploration behavior was defined as animals touching or sniffing objects within 2 cm.

### Primary hippocampal neuronal culture

On embryonic Day 18, the hippocampus was dissected from embryonic brains by using a microscope and digested with papain and DNase (DN25, Sigma). A single neuron suspension was generated by pipetting and filtering through a 40-µm nylon strainer and then plated onto plates precoated with poly-D-lysine (P1024, Sigma). The neurons were cultured in neurobasal medium (21003-049, Invitrogen) with B27 (17504-044, Invitrogen), 1% L-glutamine (25030-081, Gibco), and 0.5% penicillin/streptomycin (450-201-EL, Multicell Techs, Inc.) in an atmosphere containing 5% CO_2_ at 37 °C. The medium was changed every 3 days. After growing for 7 days, hippocampal neurons were exposed to Meth (900 µM) for 24 h.

### Cell Culture

The human embryonic kidney cell line 293T and mouse neuroblastoma N2a cell line were originally obtained from the American Type Culture Collection (ATCC). The cell line was maintained in Dulbecco’s modified Eagle’s medium (DMEM, 11,665,092, Gibco). All media were supplemented with 10% FBS (FS201-02, Transgene Biotech) and 1% penicillin and streptomycin. Cells were incubated at 37 °C in a humid 5% CO2:95% air environment.

For starvation, after aspiration of the cell culture medium and two washes with PBS, HBSS (C0219, Beyotime) medium was added and then incubated for 6 h.

### Adenoviral Vector transfection

Adenoviral vectors carrying 3 × Flag-Stx17 and EGFP-LC3 were purchased from Hanbio Biotechnology. Primary hippocampal neuron and N2a cell transfection was performed according to the manufacturer’s instructions. Thirty-six hours after transfection, the cells were incubated with the indicated reagents.

### Cell transfection

Expression vectors for Flag-tagged Stx17 were obtained from Hanbio Biotechnology. Cells were transfected with plasmids by using jetPRIME (101,000,046, PloyPlus) according to the manufacturer’s instructions. Twenty-four hours after transfection, cells were collected for analysis.

### EGF receptor trafficking assay

Primary hippocampal neurons were subjected to serum starvation conditions before stimulation with 50 ng/ml epidermal growth factor (EGF, Thermo Fisher Scientific) for various time points, then washed twice in PBS and collected for analysis.

### Immunoprecipitation

For immunoprecipitation, cell lysates or animal brains were lysed in Nonidet P-40 cell lysis buffer, 0.5 M Tris pH 7.3, 5 M NaCl, 1 M MgCl_2_, 1 M EDTA, and 80% glycerol with protease inhibitors. Cell lysates were added to prewashed magnetic beads (70,024 S, Cell Signaling Technology) and incubated with rotation for 20 min at room temperature, and then, the beads were separated from the lysates. Immunoprecipitation was performed using the indicated primary antibodies, and 1 µg of antibodies was added to 100–500 µg of cell lysate and then incubated with rotation overnight at 4 °C. Afterward, the magnetic beads were prewashed, and the lysate and antibody solution were transferred to the tube containing the prewashed magnetic bead pellet. After that, the cells were incubated with rotation for 2 h at room temperature. Beads were washed five times using lysis buffer, and the samples were boiled with SDS-containing sample buffer and finally analyzed by immunoblotting.

### GTP-agarose affinity isolation for Rab7 activity assays

GTP-Rab7 was measured using GTP agarose beads (Sigma, G9768). Cells or mouse brain tissues were collected and lysed in a buffer containing 50 mM Tris-HCl pH 7.5, 250 mM NaCl, 5 mM MgCl2, 0.5% Triton X-100, and protease inhibitors. Equal amounts of agarose beads were added to the protein lysate and then incubated with rotation overnight at 4 °C. Then, the beads were washed and suspended in 30 µl of SDS-PAGE sample buffer. Finally, the proteins were analyzed by western blotting.

### Immunoisolation of late endosomes

For immunoisolation of late endosomes, animal brains were lysed in Nonidet P-40 cell lysis buffer, 0.5 M Tris pH 7.3, 5 M NaCl, 1 M MgCl2, 1 M EDTA, and 80% glycerol with protease inhibitors. Anti-Rab7 antibody was added to prewashed magnetic beads (70,024 S, Cell Signaling Technology) and incubated with rotation for 2 h at 4 °C. Then, lysates were added to the buffer containing magnetic beads and incubated for 4 h at 4 °C. After incubation, the beads were washed 3 times and suspended in 30 µl of SDS-PAGE sample buffer. Finally, the proteins were analyzed by western blotting.

### Transmission electron microscopy (TEM)

Autophagy-related membrane structures were evaluated by transmission electron microscopy. The brains were cut into blocks (Leica UC7, Leica), fixed in stationary liquid (G1102, Servicebio), postfixed in 1% osmium tetroxide for 2 h, dehydrated in a graded series (20–100%) of ethanol, and finally polymerized with an 812 Embedding Kit (90529-77-4, SPI) for 48 h at 60 °C. Each block was observed under a transmission electron microscope (HT7700, Hitachi).

### Western blot

The cell and tissue samples were lysed in RIPA lysis solution containing protease inhibitor and phosphatase inhibitor. The protein concentration of each sample was determined using a BCA Protein Assay Kit (23,227, Thermo Fisher Scientific), and 5× loading buffer was added to each sample. The samples were boiled at 100 °C for 5 min and then subjected to separation on a 10% SDS-PAGE gradient gel (PG113, Epizyme Biotechnology). The separated proteins were transferred to polyvinylidene difluoride (PVDF, IPVH00010, Millipore) membranes, which were then blocked with TBST buffer (Tris-buffered saline with 0.1% Tween-20) containing 3% bovine serum albumin (BSA). Primary antibodies were incubated with the membranes overnight at 4 °C. After TBST washes, HRP-conjugated secondary antibodies were added to the membranes and incubated at room temperature for 2 h. The signal from each blot was captured by ECL chemiluminescence, and ImageJ was used to quantify the results from the Western blot bands.

### Tissue immunostaining

The mouse brains were fixed with 4% paraformaldehyde. Following dehydration, the tissues were immersed in OCT compound, frozen for sectioning, blocked with a blocking solution containing 5% goat serum for 30 min, and permeabilized with 0.3% Triton X-100 for 30 min at room temperature. The primary antibodies were incubated with the tissues at 4 °C overnight and then incubated with secondary antibodies for 1 h at room temperature. Following washing, the sections were mounted with DAPI (ab104139, Abcam), and the fluorescence intensity was analyzed by confocal microscopy (LSM700, Zeiss).

### Cell imaging

Cells grown on glass coverslips were fixed with 4% paraformaldehyde in PBS for 15 min, permeabilized with 0.3% Triton X-100, and blocked with 5% goat serum. After that, the cells were stained with the indicated primary antibodies at 4 °C overnight, washed with PBS, and then incubated with fluorescent dye-conjugated secondary antibodies for 2 h at room temperature. Nuclei were counterstained with DAPI, and the cells were subjected to confocal microscopy (LSM700, Zeiss). When conducting fluorescence image colocalization analysis, we utilized the FIJI software (NIH, https://imagej.net/Fiji). Using FIJI, we performed brightness and contrast adjustments on the acquired multi-channel images and separated them into individual channels. Background subtraction was applied to each channel to minimize the impact of background signals on the results. Appropriate brightness thresholds were set in each channel to clearly separate the signal from the background, ensuring accurate detection of signal regions. The “Analyze Particles” tool in FIJI was employed for comprehensive analysis of fluorescence signals in each channel. Parameters were adjusted to ensure precise measurement of colocalization areas. Further analysis was conducted using the Coloc 2 plugin in FIJI to quantify the degree of colocalization, including the interaction between Stx-17 and other markers. Finally, we exported the analysis results, including the percentage of colocalization and relevant images. This approach ensures that our observed fluorescence colocalization effects are presented in a visual and quantitative manner in the study.

### Enzyme-linked immunosorbent assay (ELISA)

The Aβ_42_ levels in mouse brain tissues were quantified by ELISA kits (Elabscience, E-EL-R1402c) according to the manufacturer’s instructions. Briefly, 100 µl of standards or samples were added to each well. Immediately, 25 µl of biotinylated corticosterone was added and then incubated for 1 h at 37 ℃. Then, 100 µl of the streptavidin-peroxidase conjugate was added to each well, and the plate was incubated for 30 min at 37 ℃. After washing, 90 µl of chromogen substrate was added and incubated for 15 min under the same conditions. The optical density (O.D.) of Aβ_42_ was measured at 450 nm using a plate reader immediately after the reaction was terminated by adding 50 µl of the stop solution.

### Quantitative RT‒PCR

Total RNA was extracted from cell lysates using TRIzol (343,903, Life Technologies) reagent according to the manufacturer’s instructions. A NanoDrop spectrophotometer (NanoDrop 2000, Thermo) was used to determine the quantified total RNA. Relative mRNA levels were detected using SYBR Green Supermix (11201ES08, Yeasen Biotech Co., Ltd.). Changes in fluorescence were monitored on a LightCycler 480 (Roche). GAPDH was used as the internal control. The sequences of the primers used for RT‒PCR are listed in Table 1.

**Table 1:** mRNA primer sequences

	Primer sequence (5’to 3’)	Primer sequence (5’to 3’)
Rabex5	ACAGATGGGCAGGTTCC	CAGGCACAGATGTAGGCA
Rin1	CACATTGCTCACTGGGGA	ACTCGGAGGAGGTGCTGG
Rin2	TGCCTATGGGGCTCTATC	AGTGTCCCTGGCTTCTGA
RME6	ACAGTCAGAAACACCA	TTATCAACACAAACACCA
Stx17	AGAGCTGCTGCAAGAGGAAG	TGCACCGCTGATACTTCTCG
GAPDH	AAGAAGGTGGTGAAGCAGG	GAAGGTGGAAGAGTGGGAGT

### Statistical analysis

Each experiment was performed at least 3 times, and all data are presented as the means ± standard error of the mean (SEM). Statistical analyses were performed by a two-tailed unpaired t test. The differences between groups were determined by one-way ANOVA with a Bonferroni correction using Prism (GraphPad v.9.0.1), and *p* < 0.05 indicated significance.

## Results

### Effects of Stx17 on cognitive and spatial learning after Meth exposure in mice

**Fig. 1:**
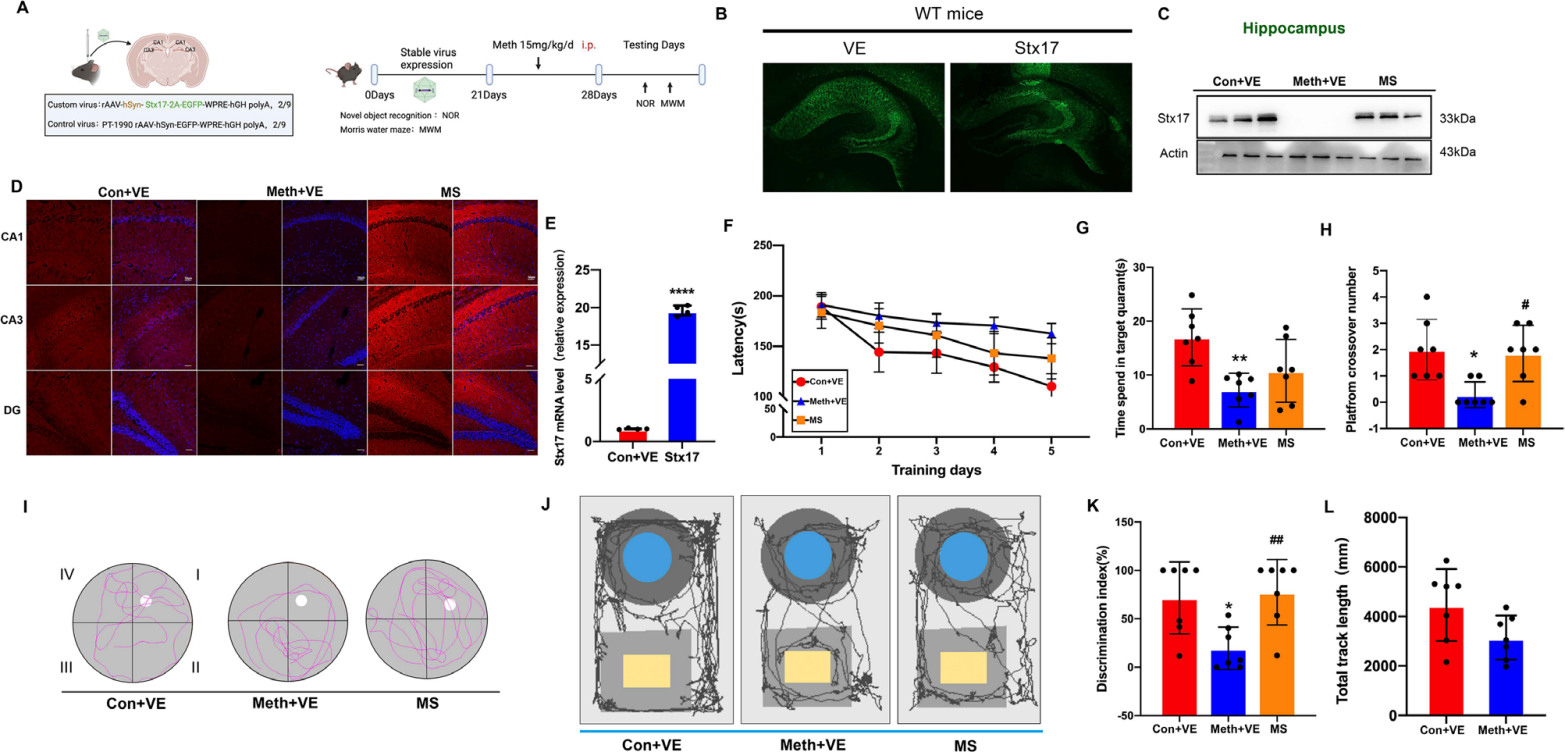
Effects of Stx17 on cognitive and spatial learning after Meth exposure in mice. **(A)** Schematic representations of experimental protocols. Bilateral AAV injection sites in the hippocampal CA1 and CA3 regions. **(B)** Representative images of immunochemical staining in the hippocampal CA1 and CA3 regions after control AAV and Stx17-AAV injection. **(C)** Twenty-eight days after control AAV and Stx17-AAV injection and Meth exposure, Stx17 levels in the hippocampus of mice were determined by Western blot analysis. **(D)** Hippocampal CA1, CA3 and DG stained with anti-Stxin17 antibody (red) and DAPI (blue), scale bar = 50 μm. **(E)** Statistical graph of stx17 mRNA level by PCR after control AAV and Stx17-AAV injection. Data are shown as the mean ± SEM (*n* = 5). ^****^p < 0.0001 compared with the control group. **(F)** After Meth exposure and Stx17-AAV injection, escape latency was measured as the mean time during training days in the Morris water maze (MWM). **(G)** Time spent in the target quadrant in the navigation test. Data are shown as the mean ± SEM (*n* = 7). ^**^p < 0.01 compared with the control. **(H)** The number of platform crossings in the probe test. Data are shown as the mean ± SEM (*n* = 7). ^*^p < 0.05 compared with the control group; ^#^*p* < 0.05 compared to the Meth group. **(I)** Representative swim paths during the probe trial. **(J)** Representative tracks during the novel object recognition test (NOR). **(K)** The discrimination index (DI) of mice in the novel object recognition test (NOR) was calculated for each experimental group. Data are shown as the mean ± SEM (*n* = 7). ^##^*p* < 0.01 compared to the Meth group. **(L)** Total distance moved by mice in the novel object recognition test (NOR). Data are shown as mean ± SEM (*n* = 7)

Our previous work indicated that Meth induced a dramatic reduction of Stx17 in primary neurons (Xu et al. 2021, Zhu et al. 2022). Surprisingly, the expression of Stx17 in the hippocampus almost vanished in the Meth-treated mice. For analysis of the potential roles of Stx17 in the regulation of cognitive behaviors and neuropathological changes following Meth exposure, AAV-Stx17 and the corresponding control plasmids were stereotaxically injected into the hippocampal CA1 and CA3 regions of C57/B6 mice. After Stx17 overexpression, mice were intraperitoneally administered Meth at a dose of 15 mg/kg/d for one week (Fig. 1A-E), and then, spatial memory tests were performed.

The Morris water maze (MWM) test showed that the mice in the Meth group exhibited a longer latency than those in the control group, an effect that was substantially reversed by AAV-Stx17 injection (Fig. 1F). In the probe trial, the mice in the Meth-treated group spent a significantly lower percentage of time in the target quadrant, whereas overexpression of Stx17 in the hippocampus partially rescued the Meth-induced cognitive impairment, resulting in more time spent in the target quadrant compared with that of the Meth group (Fig. 1G). Moreover, the number of platform crossings was evaluated. After the platform was removed, the number of platform crossings in the Meth group was significantly reduced, a phenomenon that was improved in the Stx17-overexpressing mice (Fig. 1H-I). Moreover, the novel object recognition (NOR) assay was performed to evaluate short-term memory in mice. As depicted in Fig. 1J and K, the discrimination index of the Meth group was significantly reduced compared with that of the control group, and this effect was markedly mitigated in the Stx17-overexpressing mice (Fig. 1J-K). Meanwhile, the total distance traveled was not significantly different between the two groups of mice (Figure 1L). These results suggest that Stx17 in the hippocampus exerts a salutary effect on cognitive and spatial learning against Meth-induced memory impairments.

### Effects of Stx17 on pathological and synapse-related protein expression after Meth exposure

**Fig. 2:**
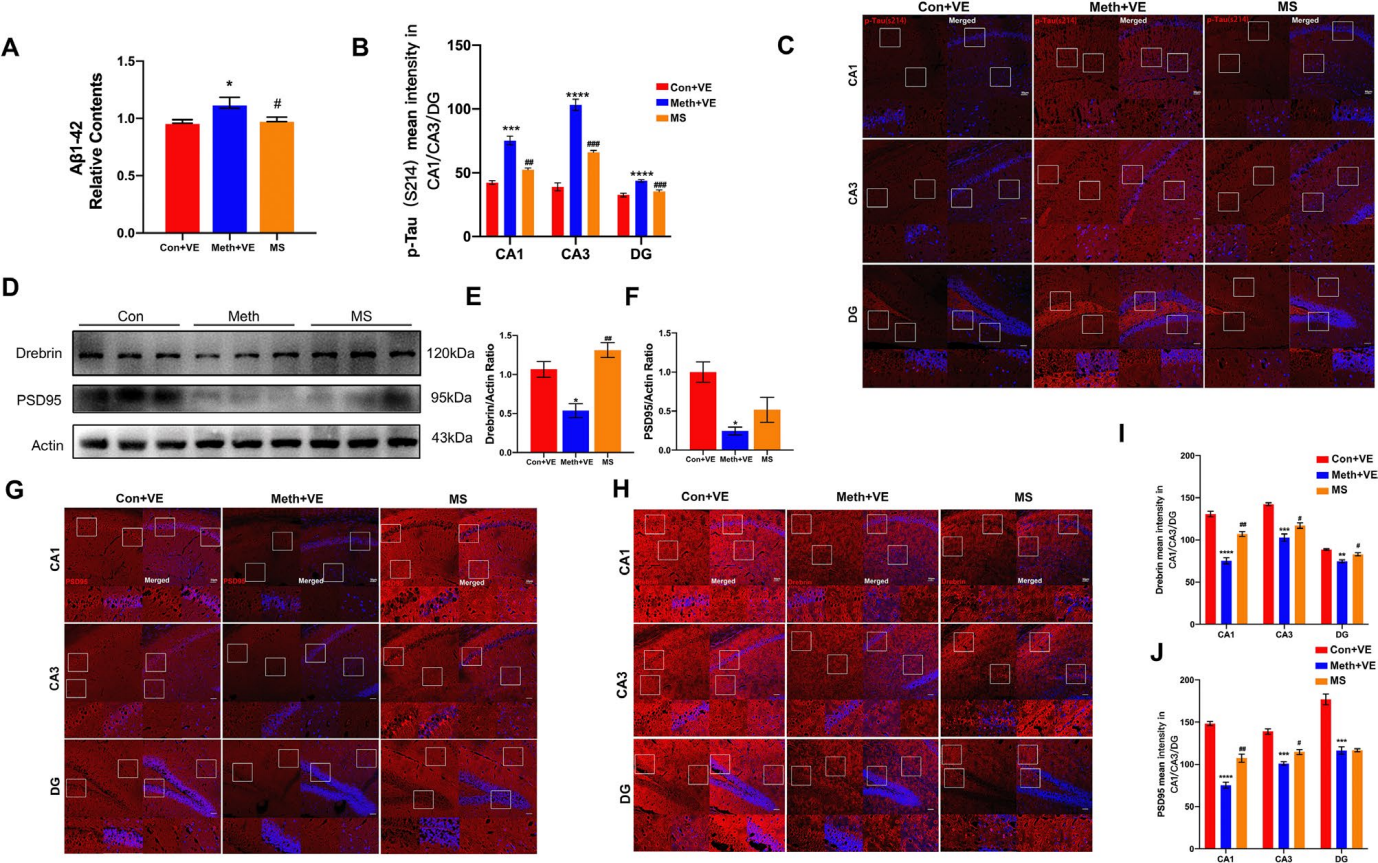
Effects of Stx17 on pathological and synapse-related protein expression after Meth exposure. **(A)** Intracellular Aβ_1−42_ in the mouse hippocampus was measured by ELISAs. Data are shown as the mean ± SEM (*n* = 3). ^*^p < 0.05 compared with the control group; ^#^*p* < 0.05 compared to the Meth group. **(B, C)** Hippocampal CA1, CA3 and DG stained with anti-p-tau (s124) antibody (red) and DAPI (blue), scale bar = 50 μm. **(D-F)** The levels of Drebrin and PSD95 in the hippocampus were assessed by western blotting after Meth exposure and Stx17 overexpression. Data are shown as the mean ± SEM (*n* = 3). ^*^p < 0.05 compared with the control group; ^##^*p* < 0.01 compared to the Meth group. **(G-J)** Hippocampal CA1, CA3, and DG regions stained with anti-Drebrin antibody (red), anti-PSD95 antibody (red), and DAPI (blue); scale bar = 50 μm. Data are shown as the mean ± SEM (*n* = 3). ^***^p < 0.001 and ^****^p < 0.0001 compared with the control group; ^#^*p* < 0.05 and ^##^*p* < 0.01 compared to the Meth group

Accumulating evidence suggests that Meth exposure aggravates Aβ synthesis and elevates p-tau levels (Shukla et al. 2019; Xu et al. 2018; Ding et al. 2020). Thus, we investigated whether Stx17 overexpression decreased the Aβ plaque load and the expression level of p-tau in the CA1, CA3, and DG regions of the hippocampus. ELISAs showed that Meth elicited a pronounced increase in Aβ, and as expected, the increased Aβ in the Meth-treated mice was significantly ameliorated in the Stx17-overexpressing mice (Fig. 2A). Moreover, the fluorescence intensity of p-tau (S214) was significantly enhanced in the hippocampal CA1, CA3, and DG regions of the Meth-treated group, while after Stx17 overexpression, the p-tau (S214) fluorescence intensity was strikingly diminished compared with that in the Meth group (Fig. 2B-C). Given the deleterious effects of Meth on cognition and memory in mice, the synaptic biomarkers PSD95 and Drebrin were examined, since these two proteins are closely related to neuronal synaptic plasticity, learning and memory (Maiti et al. 2021). The postsynaptic proteins Drebrin and PSD95 in the Meth-treated group obviously decreased, consistent with the salutary effects of Stx17 on Meth-induced impairment of cognition and memory, Stx17 overexpression in the mouse hippocampus impeded the decreased levels of Drebrin and PSD95 (Fig. 2D-J). Collectively, Stx17 overexpression in hippocampal neurons mitigates Meth-induced neurodegenerative changes, underpinning the importance of Stx17 in the protection against Meth-associated neurotoxicity.

### Stx17 overexpression in the hippocampus alleviates autophagic defects

**Fig. 3:**
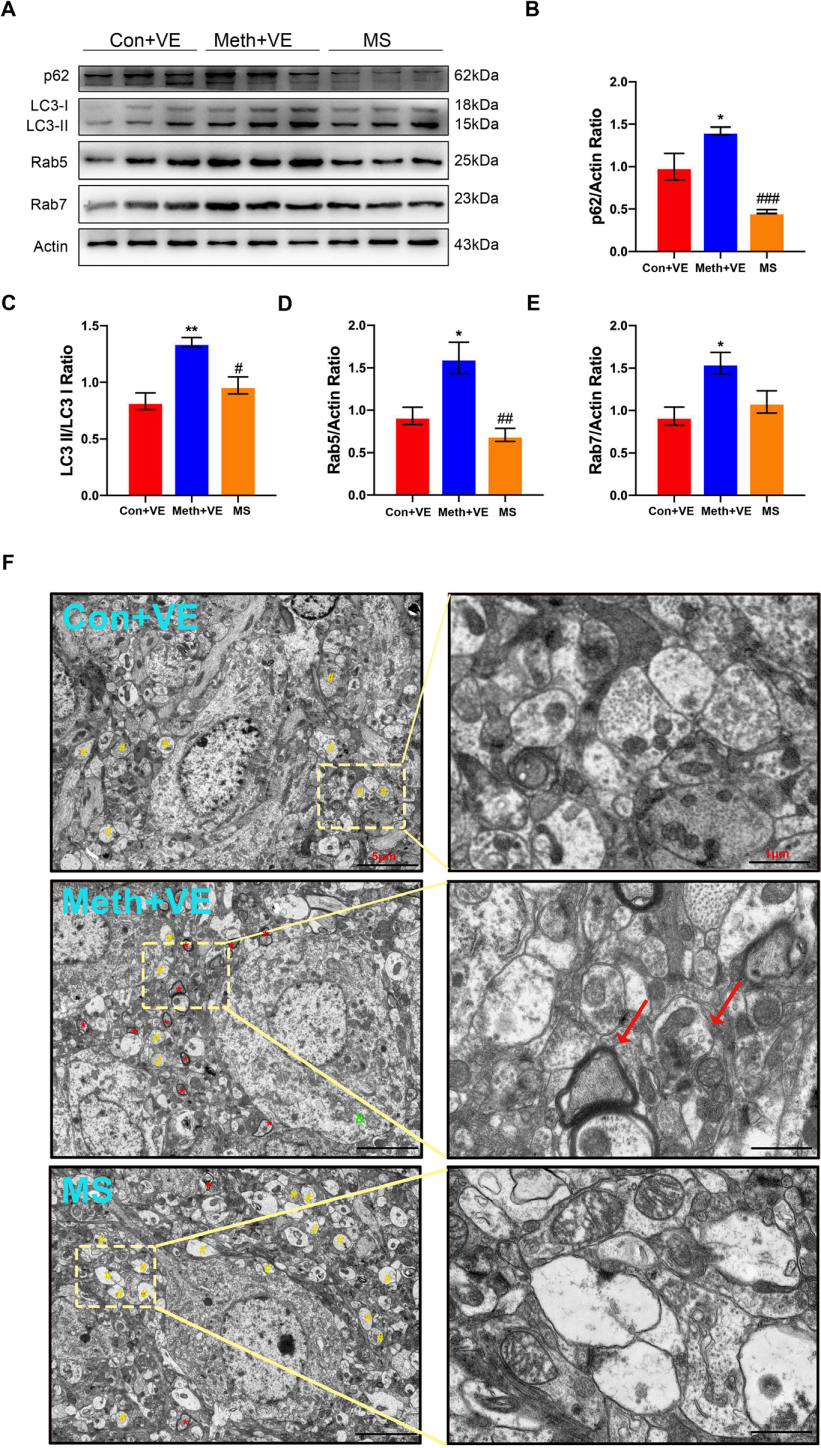
Stx17 overexpression in the hippocampus alleviates autophagic defects. **(A-E)** p62, LC3II/I, Rab5, and Rab7 levels in the hippocampus were determined by Western blotting after Meth exposure and Stx17 overexpression in mice. Data are shown as the mean ± SEM (*n* = 3). ^*^p < 0.05 and ^**^p < 0.01 compared with the control group; ^#^*p* < 0.05 and ^##^*p* < 0.01 compared to the Meth group. **(F)** Autophagy-related membrane structures were observed in the hippocampus in Meth-exposed and Stx17-overexpressing mouse brains by transmission electron microscopy. ^*^ and red arrows represent autophagosomes with double- or multilayer membrane structures, and # stands for autophagolysosomes with a monolayer membrane structure. Scale bar = 5 μm

Since Stx17 overexpression exerts a protective effect against the aforementioned Meth-induced neuronal damage, we investigated whether Stx17 exerts beneficial effects on the autophagic process. The levels of the autophagic substrate p62, autophagy marker LC3, and endosomal markers Rab5 and Rab7 in the hippocampus were detected. As depicted in Fig. 3, the ratio of LC3II/LC3I and the levels of p62, Rab5 and Rab7 in the Meth-treated group were significantly increased compared with those in the control group; however, these processes were substantially attenuated by Stx17 overexpression (Fig. 3A-E). To address the Meth-induced autophagic defect, we performed transmission electron microscopy (TEM) to examine the subcellular structures in the mouse hippocampus. Compared with the control, Meth treatment elicited a striking edema of the cell body, along with numerous autophagosomes with double- or multilayer membrane structures, which cannot fuse with the lysosomes, indicating the accumulation of vesicles and defects in cargo and autophagic substrate degradation. Notably, overexpression of Stx17 in hippocampal neurons remarkably restored the Meth-induced subcellular structure impairments, manifesting as recovered autophagolysosomes with a monolayer membrane structure (Fig. 3F).

### Time course expression of autophagy-related proteins after Meth exposure in primary hippocampal neurons

**Fig. 4:**
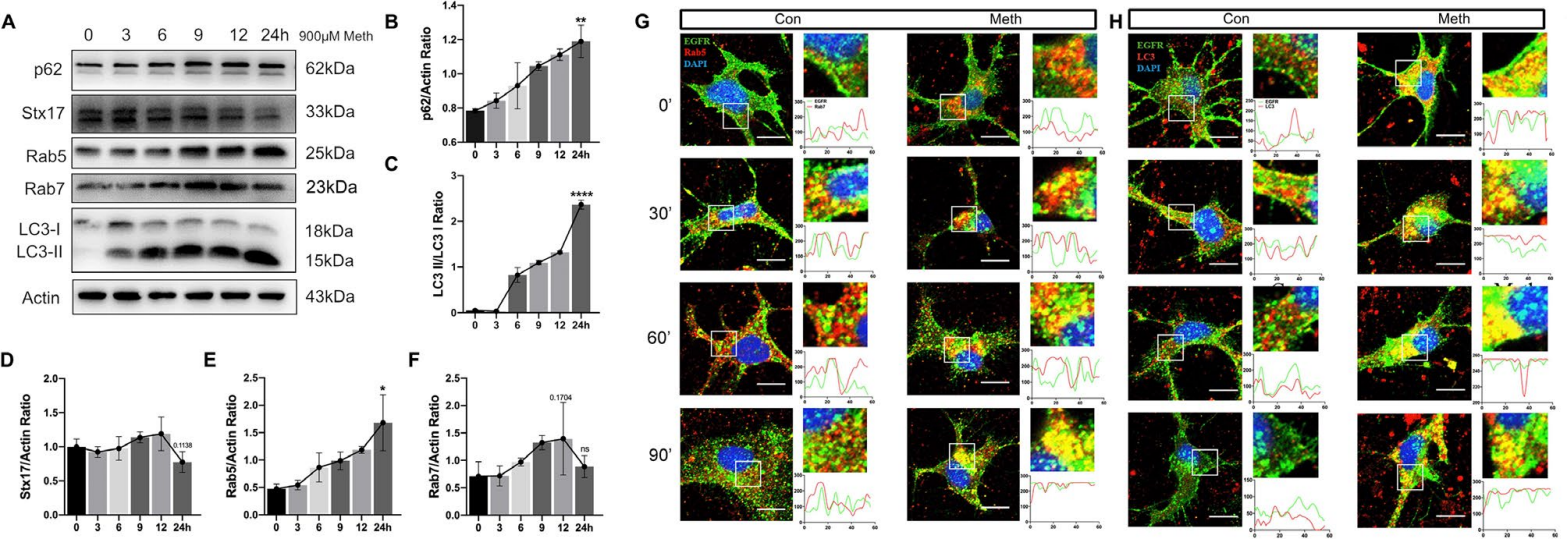
Time course expression of autophagy-related proteins and EGFR accumulation in early endosomes and autophagosomes after Meth exposure in primary hippocampal neurons. **(A-F)** After Meth exposure at different doses, the levels of p62, Stx17, Rab5, Rab7, and LC3II/I were assessed by Western blot analysis in primary hippocampal neurons. Data are shown as the mean ± SEM (*n* = 3). **(G-H)** EGF receptor trafficking assay. After Meth exposure, primary hippocampal neurons were costained with anti-EGFR (green), anti-Rab5 (red), or anti-LC3 (red) antibodies and observed by immunofluorescence, along with a statistical graph. Scale bar = 5 μm

The defects in autophagy were further validated in an in vitro study. To determine the time course of Meth action, we incubated hippocampal neurons with 900 µM Meth for 0 to 24 h, and the levels of autophagy-associated proteins were examined. Consistent with the results represented in the hippocampus, the ratio of LC3-II/LC3-I and p62 increased in neurons, with a peak response at 24 h. Additionally, Rab5, an early endosomal marker, increased after Meth exposure with a maximal response at 24 h; interestingly, Rab7, the late endosomal marker, increased after Meth exposure and decreased steadily after 12 h. The overall changes in Stx17 expression fluctuated after Meth exposure with a decreasing tendency and stable decrease after 12 h (Fig. 4A-F). The time-course action of Meth on Rab7 was similar to that on Stx17, indicating that late endosomal maturation might be related to Stx17.

### Meth exposure causes EGFR accumulation in early endosomes and autophagosomes in primary hippocampal neurons

To assay the accumulation of the cargo and substrate, we examined the trafficking of epidermal growth factor receptor (EGFR), a model receptor deemed to be degraded in lysosomes. After stimulation with EGF for 30 min, the receptor was enriched in Rab5-positive early endosomes and LC3-positive autophagosomes in both the control and Meth-treated neurons, indicating intact internalization. After 90 min, the colocalization of EGFR with Rab5-positive endosomes and LC3-positive autophagosomes in the control neurons was significantly reduced, indicating efficient EGFR degradation from early endosomes and autophagosomes. Intriguingly, EGFR was obviously trapped in Rab5-positive vesicles and LC3-positive autophagosomes in the Meth-treated neurons over time (Fig. 4G-H), suggesting that Meth exposure leads to inefficient degradation of EGFR in lysosomes.

### Stx17 overexpression ameliorates the inhibition of autophagosome maturation induced by Meth

**Fig. 5:**
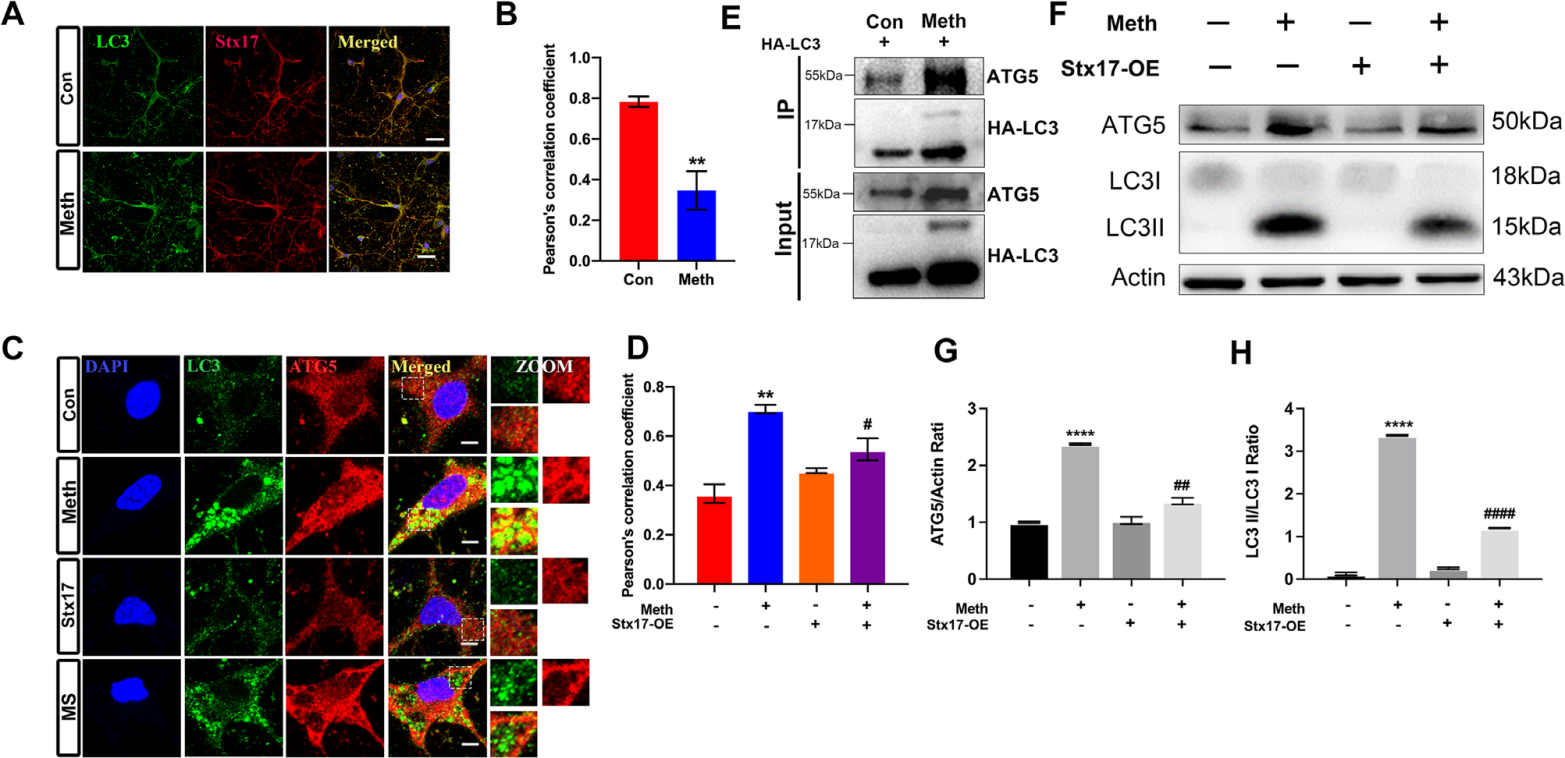
Stx17 overexpression ameliorates the inhibition of autophagosome maturation induced by Meth. **(A-B)** Colocalization of Stx17 and LC3 in primary hippocampal neurons after Meth exposure was observed by immunofluorescence, along with its statistical graph. Data are shown as the mean ± SEM (*n* = 3). ^**^p < 0.01 compared with the control group. **(C-D)** The effect of Meth exposure and Stx17 overexpression on the colocalization of LC3 and ATG5 in primary hippocampal neurons was observed by immunofluorescence, along with its statistical graph. Data are shown as the mean ± SEM (*n* = 3). ^**^p < 0.01 compared with the control group, ^#^*p* < 0.05 compared to the Meth group. **(E)** Immunoprecipitation analysis of interactions among HA-LC3 and endogenous ATG5 in HEK293T cells under Meth treatment. **(F-H)** The levels of ATG5 and LC3-I/II were measured by western blotting after Meth exposure and/or Stx17 overexpression. Data are shown as the mean ± SEM (*n* = 3). ^****^p < 0.0001 compared with the control group, ^##^*p* < 0.01 and ^####^*p* < 0.0001 compared to the Meth group

Since Meth treatment impairs the degradation of autophagic cargos, we investigated whether Meth has an impact on autophagosome maturation, an event essential to initiate autophagosome-lysosome fusion (Hyttinen et al. 2013; Kimura et al. 2007). Upon autophagic induction, Stx17 is recruited to the outer membrane of completed autophagosomes but not to the isolation membrane (Itakura et al. 2012; Takats et al. 2013, 2014). We then examined mature autophagosomes after Meth treatment. The immunofluorescence results showed a significant decrease in the colocalization of Stx17 and LC3 in hippocampal neurons after Meth exposure (Fig. 5A-B). Moreover, ATG5, a key protein that has been identified as an early autophagic marker located at the nascent autophagosome (Mizushima et al. 2001, 2003), was strongly colocalized with LC3. Notably, the enhanced overlap of ATG5 and LC3 mediated by Meth was strikingly reversed by Stx17 overexpression (Fig. 5C-D). These results indicated that immature autophagosome generation is induced by Meth and underpin the importance of Stx17 in vesicle maturation. Additionally, the expression of ATG5 in HA-LC3 immunoprecipitation markedly increased in the Meth-treated group (Fig. 5E). Consistent with this phenomenon, the levels of ATG5 markedly increased after Meth challenge, along with enhanced conversion from LC3I to LC3II, and Stx17 overexpression resulted in a pronounced decrease in the expression of ATG5 and the ratio of LC3-II/LC3-I (Fig. 5F-H). Taken together, these results suggest that Stx17 improves the Meth-mediated inhibition of autophagosome maturation.

### Stx17 overexpression alleviates the inhibition of endosome maturation induced by Meth

**Fig. 6:**
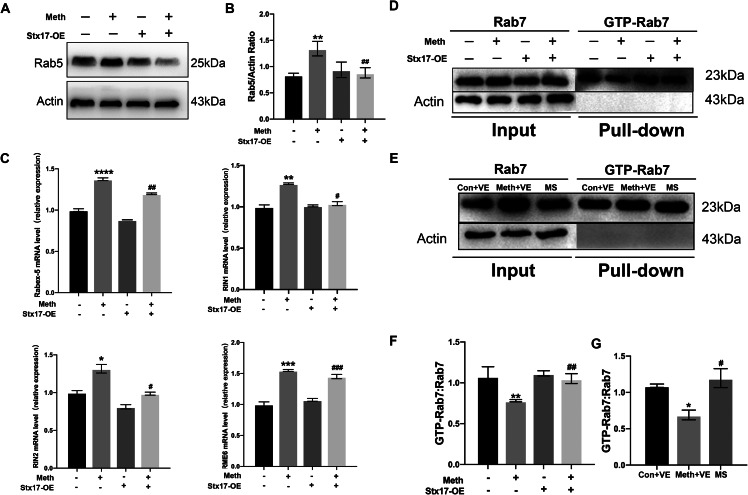
Stx17 overexpression alleviates the inhibition of endosome maturation induced by Meth. **(A-B)** The level of Rab5 was assessed by Western blot analysis in primary hippocampal neurons after Meth exposure and/or Stx17 overexpression. Data are shown as the mean ± SEM (*n* = 3). ^**^p < 0.01 compared with the control group; ^##^*p* < 0.01 compared to the Meth group. **(C)** A statistical map showing the levels of molecular markers related to Rab5 activation in primary hippocampal neurons detected by PCR after Meth exposure and Stx17 overexpression. Data are shown as the mean ± SEM (*n* = 5). ^*^p < 0.05, ^**^p < 0.01, ^***^p < 0.001 and ^****^p < 0.0001 compared with the control group; ^#^*p* < 0.05, ^##^*p* < 0.01 and ^###^*p* < 0.001 compared to the Meth group. (**D** and **F**) GTP-Rab7 in primary hippocampal neurons was determined by GTP agarose bead affinity isolation assays after Meth exposure and/or Stx17 overexpression. The “pull down” indicated affinity isolation with GTP beads. (**E** and **G**) The level of GTP-Rab7 in mouse brains after Meth treatment and AAV-Stx17 injection was determined by GTP agarose bead affinity isolation assays, and the “pull down” indicated affinity isolation with GTP beads. Data are shown as the means ± SEMs (*n* = 3). ^*^p < 0.05 and ^**^p < 0.01 compared with the control group; ^#^*p* < 0.05 and ^##^*p* < 0.01 compared to the Meth group

As the early endosome fuses into the late endosome, Rab5 is gradually replaced by Rab7. Since Meth exposure affected Rab5 and Rab7 expression, it may affect the conversion between Rab5 and Rab7, thereby retarding endosome maturation. Meth exposure led to increased levels of the early endosomal marker Rab5 and the late endosomal marker Rab7 (Figs. 3A and 4A). Given the prominent effects of Meth on Rab5 and Rab7, we further investigated whether Stx17 has an impact on endosome maturation. In accordance with our hypothesis, Meth induced a significant upregulation of Rab5 expression, an effect that was ameliorated by Stx17 overexpression (Fig. 6A-B). Since early endosome formation depends on the levels of Rab5-associated guanine nucleotide exchange factors (GEFs) (Langemeyer et al. 2018), we then examined the mRNA expression of Rab5 GEFs, including Rabex-5, RIN1, RIN2, and RME6. As expected, Meth treatment markedly increased the mRNA levels of Rabex-5, RIN1, RIN2, and RME6, whereas Stx17 overexpression impeded the elevation of these genes (Fig. 6C), indicating a potential neurotoxic effect of Meth involving immature endosome formation via Rab5 GEF, in which Stx17 plays a pivotal role in Rab5 GEF modulation.

The GTPase activity of Rab7 promotes the maturation and acidification of late endosomes; therefore, a Rab7-GTPase binding assay was performed with GTP beads to selectively bind GTP-binding proteins, and the level of GTP-bound Rab7 (Rab7-GTP) was detected in autophagic components. After endosome purification from the primary neurons and brains in mice, Meth exposure was found to selectively decrease the active form of Rab7 in endosomes, while Stx17 overexpression retarded this process, facilitating late endosome formation (Fig. 6D-G).

### Stx17 ameliorates Meth-induced autophagic dysfunction by facilitating CCZ1 activation of Rab7

**Fig. 7:**
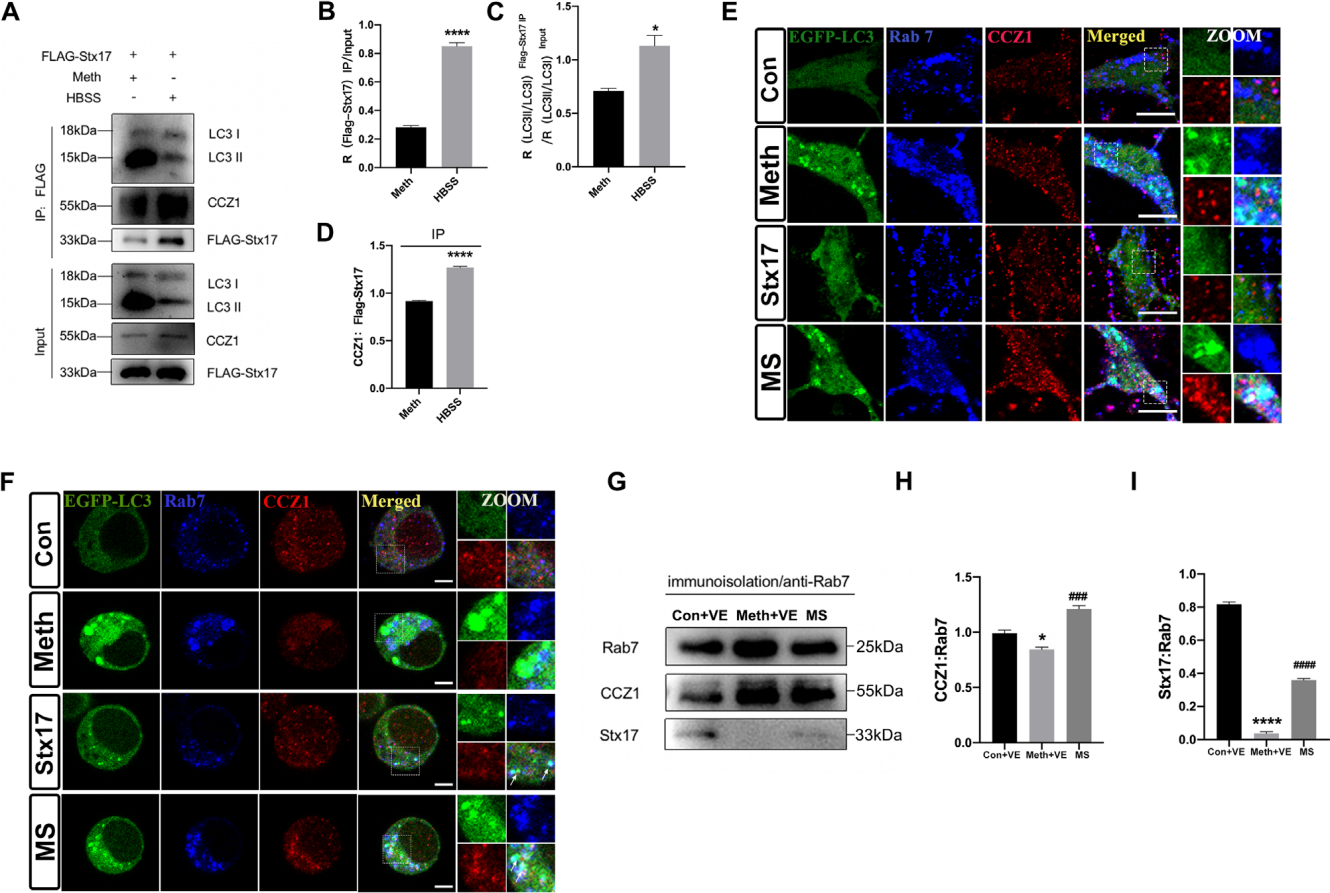
Stx17 ameliorates Meth-induced autophagic dysfunction by facilitating CCZ1 activation of Rab7. **(A)** CoIP analysis of interactions among Flag-Stx17, endogenous LC3-II/I, and CCZ1 in HEK293T cells under Meth treatment and starvation conditions. **(B)** Data indicate the means ± SEMs of the ratio between Flag-Stx17 expressed in IP and Flag-Stx17 level in inputs. Data are shown as the means ± SEMs (*n* = 3). ^****^p < 0.0001 compared with the Meth group. **(C)** Data are presented as the means ± SEMs between the LC3-II/I ratio in Flag-Stx17 IP relative (normalized) to the LC3-II/I ratio in inputs. Data are shown as the means ± SEMs (*n* = 3). ^*^p < 0.05 compared with the Meth group; **(D)** Statistical diagram of interactions between endogenous CCZ1 and Flag-Stx17. ^****^p < 0.0001 compared with the Meth group. **(E-F)** Colocalization of EGFP-LC3, Rab7, and CCZ1 in primary hippocampal neurons and N2a cells after Meth treatment and Stx17 overexpression. Scale bar = 5 μm. **(G)** The interaction between Rab7 (late endosomes) and CCZ1/Stx17 was determined by IP. Data are shown as the means ± SEMs (*n* = 3). ^*^p < 0.05 and ^****^p < 0.0001 compared with the control group; ^###^*p* < 0.001 and ^####^*p* < 0.0001 compared to the Meth group

Both forms of LC3 (LC3I and LC3II) are detected in coimmunoprecipitation with Stx17 under basal conditions, and this process can be strongly enhanced by starvation stimulation, redirecting Stx17 to LC3-II in the conventional autophagy pathway (Kumar et al. 2018). In the present study, compared to that in starvation-induced autophagy, the LC3-II/LC3-I ratio in Flag-Stx17 immunoprecipitation was reduced in the Meth-treated group, indicating that Meth exposure may lead to a decreased recruitment of Stx17 to the autophagosome membrane. Since CCZ1 functions as a GEF for Rab7 and plays a critical role in late endosome maturation (Kiontke et al. 2017; Klink et al. 2022), after Meth treatment, the expression of CCZ1 in Flag-Stx17 immunoprecipitation markedly decreased in the Meth-treated group, suggesting a reduced interaction between endogenous CCZ1 and Flag-Stx17 (Fig. 7A-D). To assess the potential effect of Stx17 on Rab7, we used EGFP-LC3 to label autophagosomes in both N2a cells and primary neurons. Immunofluorescence results showed that in the Meth-treated group, a substantial reduction in the colocalization of EGFP-LC3 with Rab7 or CCZ1 was observed; however, these phenomena could be strikingly reversed by Stx17 overexpression, represented as bright white spots of the three colocalized markers (Fig. 7E-F). In support of this event, Rab7 antibody-coated beads were used to immunopurify late endosomes from the homogenate of the adult mouse hippocampus. Compared with those of the control group, the levels of CCZ1 and Stx17 in the purified late endosomes of the Meth-treated mouse hippocampus were significantly reduced, and this effect was substantially reinstated by Stx17 overexpression (Fig. 7G-I). These findings shed light on the complex regulation of Stx17 in promoting the binding of CCZ1 to Rab7, facilitating the conversion from Rab7-GDP to Rab7-GTP, which promotes vesicle fusion.

## Discussion

Neuropathic protein accumulation was recently recognized as one of the major characteristics of Meth-induced neuronal degenerative disease (Mizoguchi et al. 2019, Zheng et al. 2022); however, the potential mechanisms remain poorly understood. however, the potential mechanisms remain poorly understood. Here, we revealed defects in cargo degradation in both in vivo and in vitro studies, elucidating Meth-induced neurogenic protein accumulation, which may be associated with the impairment of cognitive and spatial learning in mice. It is crucial to emphasize that our study contributes a novel perspective by uncovering an unrecognized mechanism mediated by Meth, with Stx17 playing a pivotal role. The presented findings highlight the intricate interplay between autophagosome/endosome maturation and vesicle-lysosome fusion in mitigating Meth-induced autophagic dysfunction, ultimately leading to improvements in memory deficits and neuropathological manifestations. Through this comprehensive exploration, we aim to provide valuable insights into the potential therapeutic strategies for neurodegenerative diseases induced by Meth, emphasizing the regulatory significance of Stx17 in the autophagic process.

Several studies have demonstrated that Meth can interfere with intracellular vesicular transport and release, leading to the accumulation of vesicles and pathological proteins in neurons (Kanthasamy et al. 2006; Lazzeri et al. 2018). We utilized the Gene Expression Omnibus (GEO) public dataset (https://www.ncbi.nlm.nih.gov/geo/) by searching for the keyword “Meth” and employing the GEO2R tool to analyze Series GSE111925, with a specific focus on the gene with ID A_55_P2174506, identified as Stx17. It revealed a significant decrease in the expression level of Stx17 in the mouse brain exposed to Meth (Figure S1A). Our previous study showed that the autophagic downstream events mediated by Stx17 decreased Stx17 levels and impaired fusion between autophagosomes and late endosomes/lysosomes, contributing to vesicular accumulation synchronously with pathological protein deposition (Xu et al. 2021; Zhu et al. 2022). In the current study, we extended our findings that Meth exposure induced abnormally increased immature vesicles in vitro and in vivo, a crucial event in the initiation of autophagy. To ascertain the impacts of immature vesicles on vesicular cargo degradation, we performed an EGFR trafficking assay, which is used to prove intracellular EGFR degradation via the lysosomal pathway (Frey et al. 2021), and showed that EGFR degradation stimulated by EGF was sharply retarded by Meth treatment. Intriguingly, EGFR was abundantly deposited in early endosomes and autophagosomes, prompting us to hypothesize that Meth might exert its effects on vesicle maturation and therefore impede autophagosome-late endosome/lysosome fusion.

There are multiple stages in the formation and decomposition of autophagosomes. Autophagosome maturation, an essential step, ensures the degradation of autophagic cargos. Stx17, a membrane protein with a hairpin transmembrane structure, comes from the cytoplasm, endoplasmic reticulum, and mitochondria. This molecule is recruited to the fully mature autophagosome membrane upon autophagic induction. When autophagy is activated, Stx17 seals the autophagosome (mature autophagosome), participating in several stages of autophagy, including autophagosome-late endosome/lysosome fusion. This evidence highlights the upstream and downstream events in autophagy during which Stx17 plays key roles. In the current study, Meth treatment elicited a pronounced reduction in the colocalization of Stx17 and LC3, and our results indicated that compared to that in starvation-induced conventional autophagy, the targeting specificity of Stx17 on the double membrane of autophagosomes was significantly reduced in Meth-induced autophagy, implying that the decrease in mature autophagosomes was mediated by Meth. Moreover, Atg12-Atg5 is limited to the precursor of the autophagosome, and the primary role of Atg12-Atg5 in this process is to stretch the isolation membrane, which is vital for the formation of autophagic vesicles. Once the completed autophagic vesicle is formed, Atg12-Atg5 separates from the autophagosomal membrane; therefore, Atg5 is specifically situated in the immature autophagosome, which is the precursor to the autophagosome (Mizushima et al. 2001, 2003; Suzuki et al. 2001). To further verify Meth-mediated immature autophagosome generation, we showed striking ATG5 aggressions in autophagosomes after Meth challenge. More importantly, Meth facilitated a remarkable increase in the overlap between ATG5 and LC3. This evidence, taken together, strongly confirmed the inhibitory effects of Meth on autophagosome maturation.

Another intriguing finding of the current study is that Meth also exerts its effects on endosome maturation. The late endosome is formed by the membrane fusion of the early endosome, which contains a variety of hydrolytic enzymes that hydrolyze and decompose the encapsulated substances in the endosome. Rab5 is one of the key factors controlling the formation and transportation of endosomes, and excessive GEF activation promotes the GTase activity of Rab5, thereby increasing the level of Rab5-GTP and promoting the formation and quantity of early endosomes (Langemeyer et al. 2018). Notably, overexpression of Stx17 not only obviously reversed the increased levels of Rab5 caused by Meth but also reversed the GEF transcription level of Rab5. However, the potential mechanisms by which Stx17 improves the maturation of autophagosomes and endosomes and reduces neuronal damage induced by Meth need to be further clarified. Moreover, Rab7 and its GEF, which play a crucial role in promoting vesicular transport and endosomal maturation, were examined. As a Rab7 GEF, CCZ1 localizes to the autophagosome and interacts directly with Atg8 (Nordmann et al. 2010; Xian et al. 2019), facilitating the conversion of GDP-Rab7 (inactive state) to GTP-Rab7 (active state). After exposure to Meth, the activity of Rab7 decreased in both neurons and mouse brains; however, Stx17 overexpression was demonstrated to reverse this process. Research has shown that both Stx17 and CCZ1 can interact with the LC3 protein and recruit it to the autophagosome (Gao et al. 2018). Our previous research has also shown that Stx17 promotes the generation of amphisomes, which are formed by the fusion of autophagosomes and late endosomes (Xu et al. 2021; Zhu et al. 2022). In the present work, the spatial colocalization among the autophagosome, CCZ1 and Rab7 almost vanished after Meth exposure, indicating a reduction in mature endosomes and defects in amphisome formation. Intriguingly, these effects were substantially ameliorated by Stx17 overexpression. To gain a better understanding of Stx17’s regulatory role in the maturation of late endosomes, we performed the immunoisolation and purification of these endosomes from adult mouse hippocampal homogenates with Rab7 antibody-coated magnetic beads. In addition, a Co-IP assay was performed to confirm the interactions between Stx17 and CCZ1 that were triggered by Meth. As expected, a significant increase in the binding of CCZ1 to late endosomes was observed following Stx17 overexpression. These findings highlight the crucial role of Stx17 in mediating the binding of CCZ1 to endosomes, thereby revealing its critical role in endosome maturation. However, there are still several limitations in the current work. First, the exact mechanism by which Stx17 regulates the conversion of Rab5 to Rab7 remains unclear. It is uncertain whether these effects occur through direct interactions with CCZ1, indirect interactions with ATG8 and CCZ1, or other unknown regulators. Second, the role of Stx17 in regulating the GEF of Rab5 remains poorly understood. Therefore, more investigations are required to determine how Stx17 influences the activation of Rab5 GEF and the endosomal maturation process.

## Conclusions

**Fig. 8:**
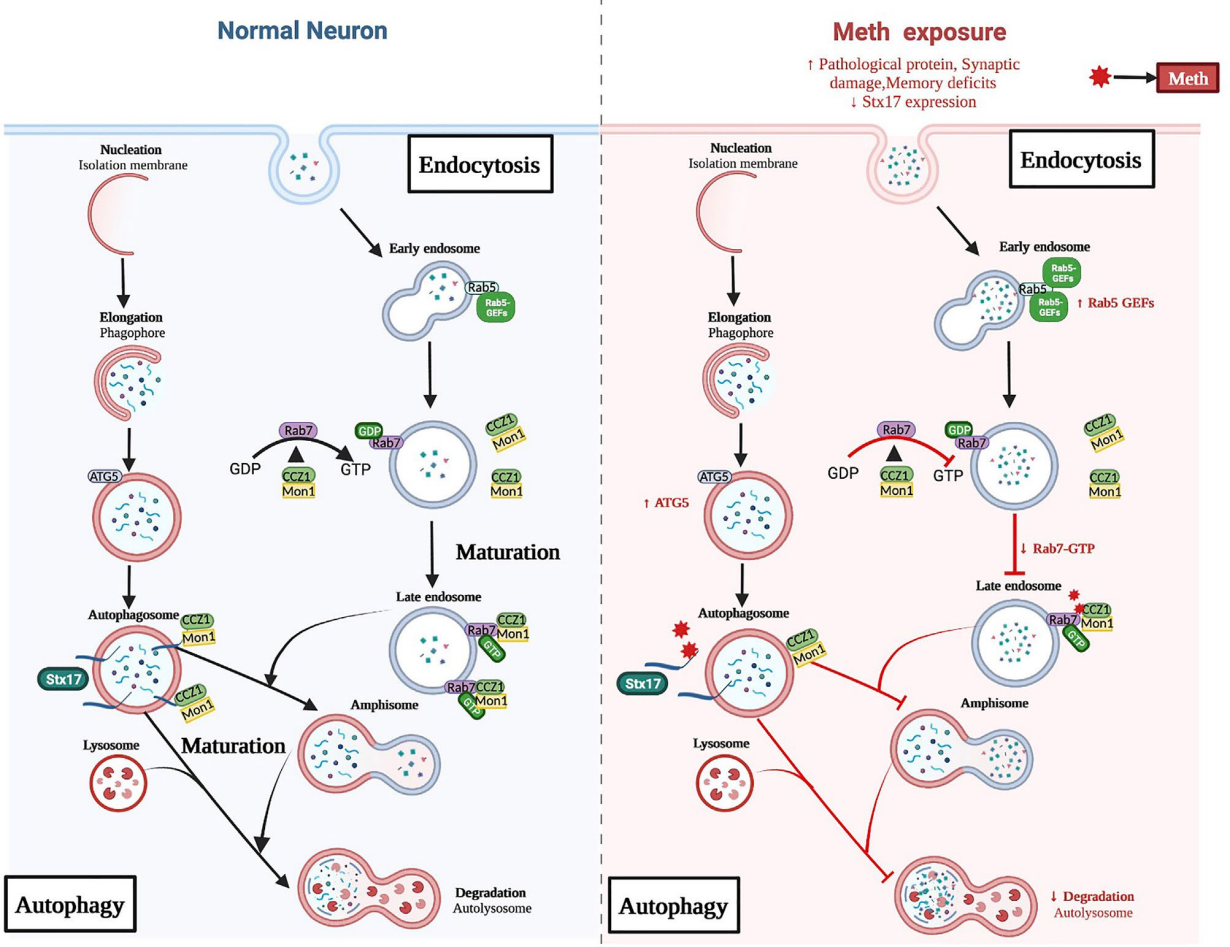
Meth-induced inhibition of autophagosome and endosome maturation mechanism schematic. Stx17 overexpression promotes CCZ1 activation of Rab7, facilitating late endosome formation and decreasing the overlap between ATG5 and LC3, promoting autophagosome maturation. These processes initiate upstream autophagosome-late endosome/lysosome fusion, alleviate Meth-induced autophagic dysfunction, and ameliorate memory impairment and neuropathology

In conclusion, our study provides evidence for the role of Stx17 in regulating autophagosome and endosome maturation and partially deciphers the beneficial effects of Stx17 on Meth-induced neuropathological and behavioral deficits. Mechanistically, Stx17 overexpression not only promotes CCZ1 activation of Rab7, i.e., facilitating late endosome formation but also decreases the overlap between ATG5 and LC3, promoting autophagosome maturation (Fig. 8). These processes, taken together, initiate upstream autophagosome-late endosome/lysosome fusion, alleviate Meth-induced autophagic dysfunction, and ultimately ameliorate memory impairment and neuropathology. Therefore, our work underscores the importance of Stx17 in regulating autophagy and provides insight into potential therapeutic strategies for Meth-induced neurodegenerative diseases.

In terms of potential applications, our research outcomes offer promising directions for future studies and treatments. Firstly, a deeper understanding of Stx17’s regulation of key molecules in the autophagic process presents new targets for developing drugs to treat Meth-induced neuropathology. Secondly, our findings highlight the crucial role of autophagy in neurodegenerative diseases, laying the foundation for designing more precise treatment strategies. Research in these areas is expected to provide robust support for the clinical application of treatments for methamphetamine-related neurodegenerative diseases in the future.

### Electronic supplementary material

Below is the link to the electronic supplementary material.

**Supplementary Material 1**: **Fig. S1(A)** Geo database gene difference analysis. **(B)** Searching protein interaction in BioGRID database

## Data Availability

The datasets used and/or analysed during the current study are available from the corresponding author or on reasonable request.

## List of Abbreviations

Meth: Methamphetamine
Stx17: Syntaxin17
GEF: Guanine-nucleotide exchange factor
AV: Adenovirus
AAV: Adeno-associated virus
ATSs: Amphetamine-type stimulants
AD: Alzheimer’s disease
PD: Parkinson’s disease
ALS: Amyotrophic lateral sclerosis
HSP: Hereditary spastic paraplegia
EGF: Epidermal growth factor
EGFR: Epidermal growth factor receptor
MWM: Morris water maze
NOR: Novel object recognition
TEM: Transmission electron microscopy
Rab7-GTP: GTP-bound Rab7
