# Supplementary figures and images for: Key roles of autophagosome/endosome maturation mediated by Syntaxin17 in methamphetamine-induced neuronal damage in mice
Source: Mol Med. 2024 Jan 3;30:4. doi: 10.1186/s10020-023-00765-9 (PMC10765725; doi:10.1186/s10020-023-00765-9)

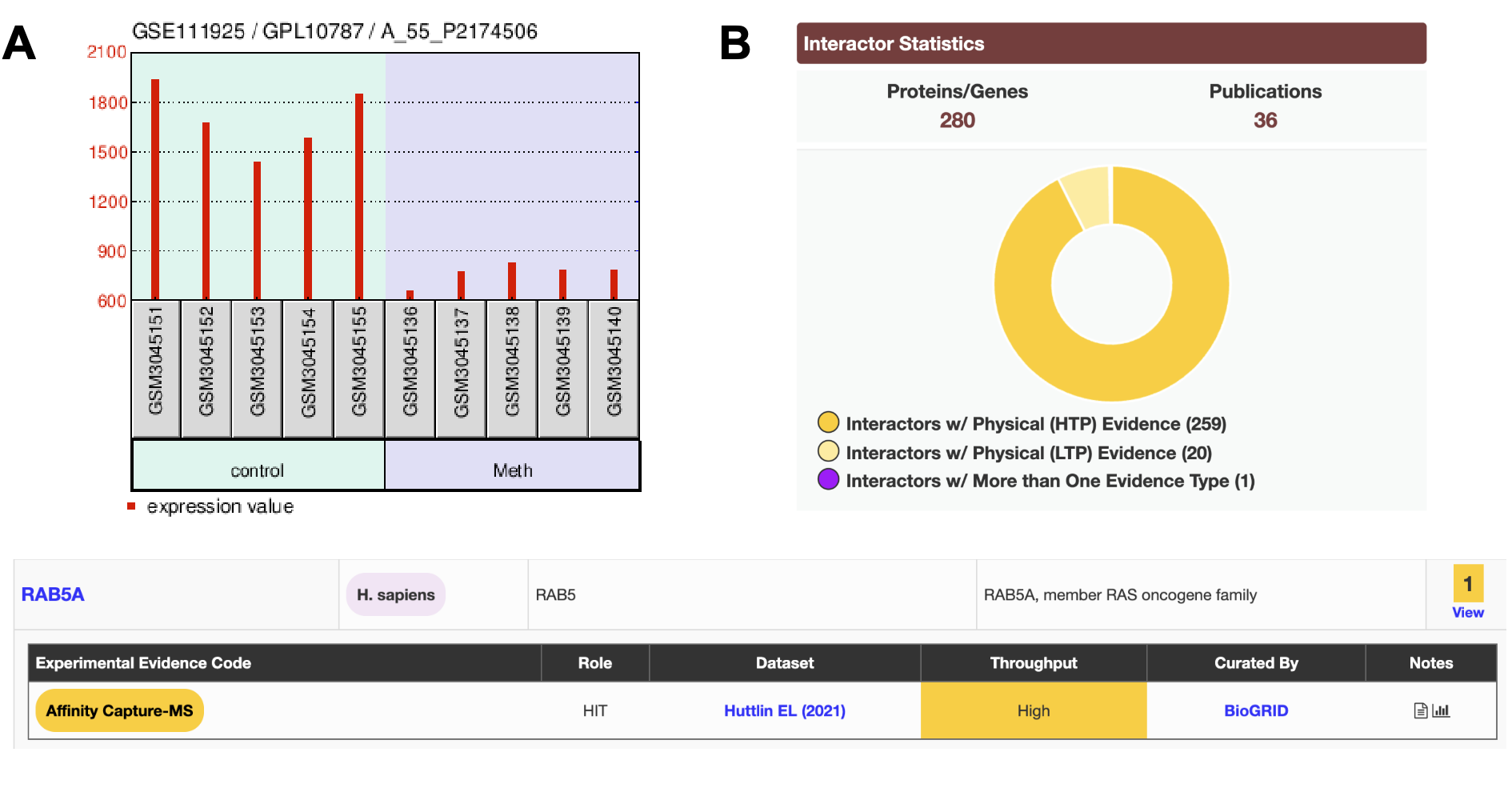

Supplement: Supplementary file 1 — Supplementary Material 1: Fig. S1(A) Geo database gene difference analysis. (B) Searching protein interaction in BioGRID database [file 10020_2023_765_MOESM1_ESM.png]
